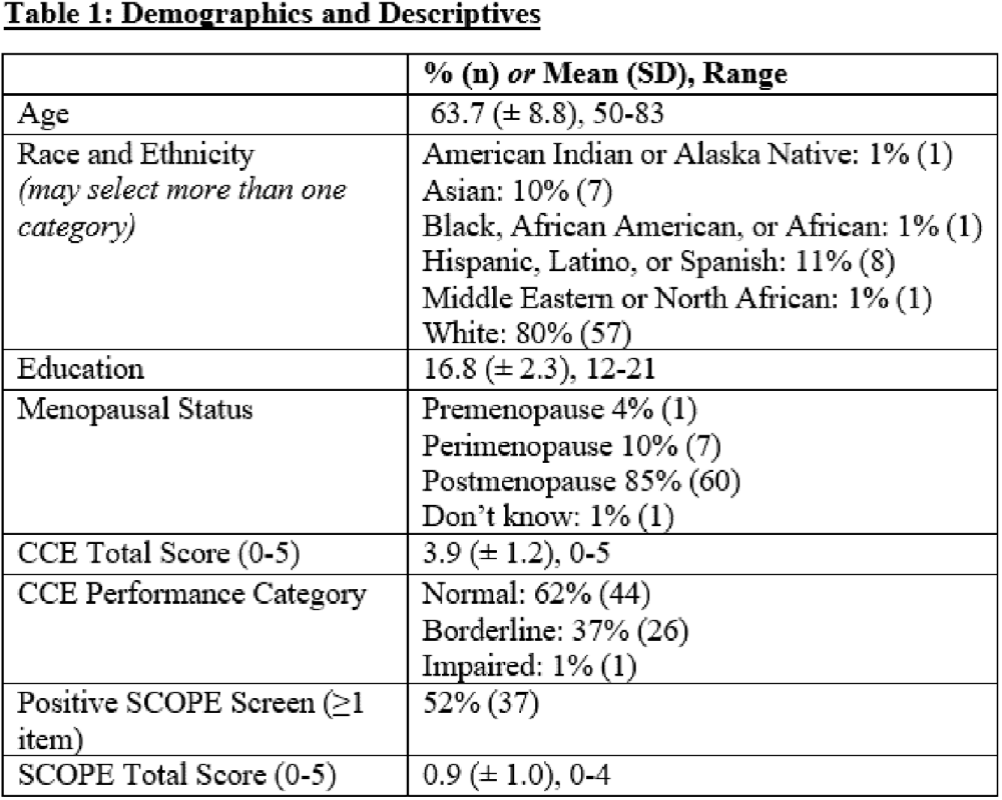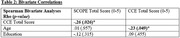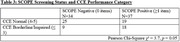# Preliminary Findings from EMPOWER: Evaluating Memory as Part of Women’s Routine Care

**DOI:** 10.1002/alz.092824

**Published:** 2025-01-03

**Authors:** Jillian L. Joyce, Stephanie Cosentino, Sandra Rizer, Samantha A. Dargie, Silvia Chapman, Laura Mora, David J. Libon, Sarah E. Tom, Karen Marder, Mary Rosser

**Affiliations:** ^1^ University of Southern California, Los Angeles, CA USA; ^2^ Columbia University, New York, NY USA; ^3^ Columbia University Irving Medical Center, New York, NY USA; ^4^ Rowan University, Stratford, NJ USA

## Abstract

**Background:**

Despite evidence that Alzheimer’s disease (AD) related pathological changes occur earlier in women than men, women are diagnosed later. To address this care disparity, the Evaluating Memory as Part of Women’s Routine Care (EMPOWER) program was established to integrate cognitive screening into routine gynecological well‐woman visits. Recent work has demonstrated the feasibility of conducting subjective cognitive screening at the well‐woman visit. The extent to which such subjective screening maps onto objective cognitive screening is critical. Here we outline preliminary results from the EMPOWER program, where both a subjective memory screener and a novel digital cognitive assessment were used to screen cognition.

**Method:**

Women 50 and older appearing for routine gynecological well‐woman visits at the Columbia University Integrated Women’s Health Center were eligible to participate in this study. Participants completed a subjective cognitive survey, the Screener for Cognitive Problems in Everyday Life (SCOPE), as well as a digital cognitive task, the Linus Health Core Cognitive Evaluation (CCE). The SCOPE scores range from 0‐5, and endorsement of any item is considered a positive screen. The CCE comprises a digitized version of the Mini‐Cog (three‐word recall and clock‐drawing). CCE performance ranges from 0‐5 points, with 4‐5 as Normal, 2‐3 as Borderline, and 0‐1 as Impaired.

**Result:**

Of 130 eligible women, 71 (55%) were recruited. Table 1 provides descriptive demographic and cognitive data. Total number of items endorsed on the SCOPE was associated with total CCE performance (Table 2). Additionally, women that screened SCOPE positive were more likely to perform in the borderline/impaired category of the CCE (Table 3).

**Conclusion:**

Integrating cognitive screening into the routine gynecological well‐woman exam can identify midlife and older women experiencing cognitive difficulties, a critical step in addressing the care disparity. The current study demonstrates the validity of a subjective cognitive screener for detecting early cognitive dysfunction. A limitation of the study is the lack of ethnic and racial diversity, especially groups that are at increased risk for AD. Ongoing work is expanding EMPOWER to clinics that serve a more sociodemographically diverse patient population. Future work should include longitudinal monitoring to assess both subjective and objective cognitive changes over time.